# Vector Transmission of *Leishmania* Abrogates Vaccine-Induced Protective Immunity

**DOI:** 10.1371/journal.ppat.1000484

**Published:** 2009-06-19

**Authors:** Nathan C. Peters, Nicola Kimblin, Nagila Secundino, Shaden Kamhawi, Phillip Lawyer, David L. Sacks

**Affiliations:** Laboratory of Parasitic Diseases, National Institute of Allergy and Infectious Diseases, National Institutes of Health, Bethesda, Maryland, United States of America; Imperial College London, United Kingdom

## Abstract

Numerous experimental vaccines have been developed to protect against the cutaneous and visceral forms of leishmaniasis caused by infection with the obligate intracellular protozoan *Leishmania*, but a human vaccine still does not exist. Remarkably, the efficacy of anti-*Leishmania* vaccines has never been fully evaluated under experimental conditions following natural vector transmission by infected sand fly bite. The only immunization strategy known to protect humans against natural exposure is “leishmanization,” in which viable *L. major* parasites are intentionally inoculated into a selected site in the skin. We employed mice with healed *L. major* infections to mimic leishmanization, and found tissue-seeking, cytokine-producing CD4+ T cells specific for *Leishmania* at the site of challenge by infected sand fly bite within 24 hours, and these mice were highly resistant to sand fly transmitted infection. In contrast, mice vaccinated with a killed vaccine comprised of autoclaved *L. major* antigen (ALM)+CpG oligodeoxynucleotides that protected against needle inoculation of parasites, showed delayed expression of protective immunity and failed to protect against infected sand fly challenge. Two-photon intra-vital microscopy and flow cytometric analysis revealed that sand fly, but not needle challenge, resulted in the maintenance of a localized neutrophilic response at the inoculation site, and removal of neutrophils following vector transmission led to increased parasite-specific immune responses and promoted the efficacy of the killed vaccine. These observations identify the critical immunological factors influencing vaccine efficacy following natural transmission of *Leishmania*.

## Author Summary

The generation of vaccines that protect against intracellular pathogens such as malaria, human immunodeficiency virus and leishmaniasis have met with limited success. A perplexing aspect of this failure as it relates to leishmaniasis is the knowledge that individuals typically get the disease only once, and that individuals who are experimentally infected with cultured parasites are protected against sand fly transmitted infection, thereby providing a “gold standard” for vaccine design. Many engineered, non-living vaccines have been developed to mimic the immune response observed in protected individuals and some of these have been shown to provide excellent protection against needle inoculation of *Leishmania* parasites in mice. However, very similar vaccine formulations adapted for use in people have failed to protect against natural exposure to infected sand fly bites. In the present study, we attempt to reconcile these long-standing differences, and to provide the critical correlates of immunity that will predict vaccination success against natural exposure.

## Introduction

*Leishmania* are obligate-intracellular protozoan parasites that establish infection in mammalian hosts following transmission to the skin by the bite of an infected *Phlebotomine* sand fly [1]. Different *Leishmania* species are associated with a spectrum of clinical outcomes in humans, including fatal, disseminated infection of the spleen and liver following infection with *L. donovani*, and self-curing cutaneous lesions associated with *L. major* and other cutaneous strains. Healed cutaneous lesions often result in a permanent scar that has been shown to harbor low numbers of parasites over the long term [2]. While this chronic, sub-clinical state can serve as a long-term reservoir for disease, it also maintains powerful protective immunity for the host, as individuals with healed primary lesions are highly resistant to re-infection, and complete elimination of a primary infection in animal models results in susceptibility to reinfection [3],[4]. Deliberate needle inoculation with viable parasites in a selected site, referred to as “leishmanization,” has been employed extensively as a live “vaccine” in people for generations, and is highly effective against natural exposure [5],[6],[7],[8]. However, due to reports of adverse reactions at the site of inoculation, quality control issues, and concerns over causing serious disease in immuno-compromised individuals, leishmanization has fallen out of favor [8],[9]. Employing the mouse model of *L. major* infection, numerous non-living [10],[11],[12],[13],[14],[15] and live-attenuated [13],[16],[17], or DNA-based [10],[18] vaccine formulations have been developed as alternatives to leishmanization, which in many cases have conferred relatively long-term protection against experimental needle challenge [10],[11],[12],[18]. In contrast, non-living vaccines, including formulations similar to those shown to work effectively in mice against needle challenge [11],[13], have yet to confer significant protection against natural exposure in people, despite the generation of measurable cell-mediated immunity [9],[19],[20],[21],[22],[23],[24],[25],[26],[27],[28]. This contradiction between the results in humans and animal trials suggests that the correlates of vaccine efficacy developed mainly from the mouse model, namely the generation of Th1 responses and the reduction of lesion size and/or parasite number following needle challenge, may not adequately define the requirements for protection against natural transmission. Observations by Rogers et al. [29], in which vaccination with soluble leishmanial antigen plus IL-12 delayed the onset of progressive lesions following needle, but not infected sand fly challenge in BALB/c mice, support this suggestion.

In addition to the delivery of infectious stage parasites into the dermis, sand flies also deposit pharmacologically active saliva, which aids in blood feeding, and egest parasite-released glycoconjugates, which accumulate behind the mouthparts in infected flies and form a promastigote secretory gel (PSG). These molecules have been shown to enhance the severity of disease when co-administered with infectious stage parasites [30],[31],[32],[33]. We have recently reported that sand fly transmission induces a qualitatively unique inflammatory response at the localized bite site that includes a dynamic recruitment of neutrophils, and that these neutrophils markedly enhance the ability of parasites to establish primary infection [34]. Thus, an analysis of the influence of sand fly transmission on vaccine efficacy is likely to be highly relevant to the generation of a *Leishmania* vaccine that is effective in people.

## Results

### Healed primary infection protects against infected sand fly challenge

**Figure 1:**
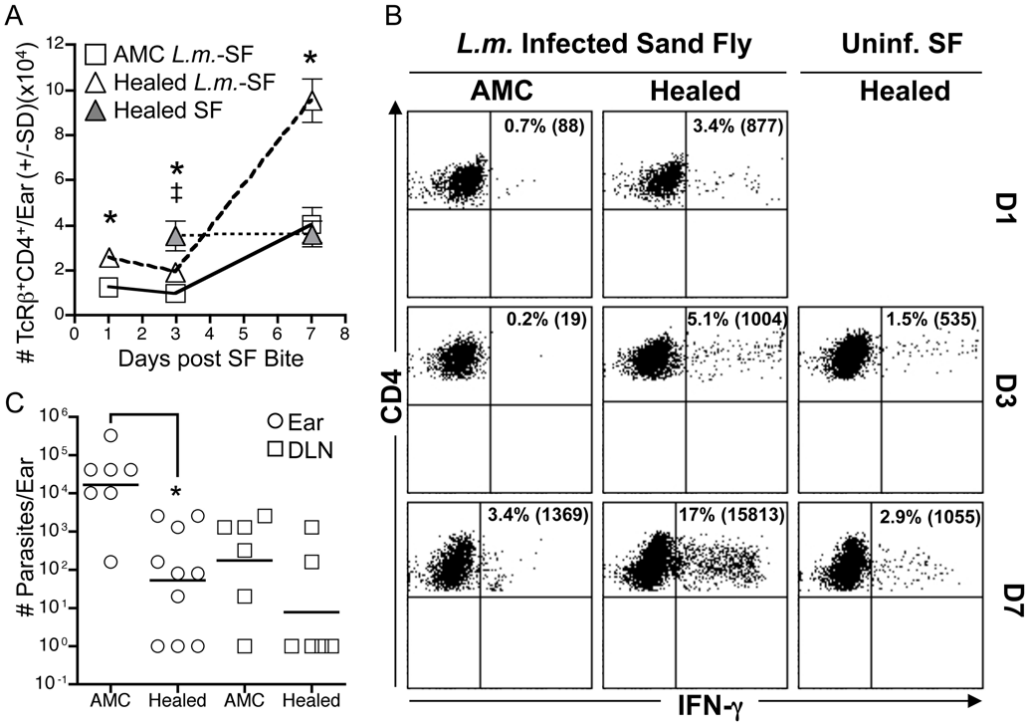
Mice with healed primary infections mount robust immunity and control parasite growth following transmission of *L. major* by infected sand fly bite. Ears of naïve, age matched control mice (AMC), or healed mice infected by needle inoculation s.c. in the footpad with 10^4^ *L.m.* metacyclic promastigotes 22 weeks previously, were exposed to the bites of 4 uninfected (SF) or *L.m.*-infected *P. duboscqi* sand flies (*L.m.*-SF). Ear derived cells were analyzed at the indicated times following exposure to sand flies. (A and B) Total number of TcRβ^+^CD4^+^ T cells per ear as determined by flow cytometric analysis of duplicate samples of pooled ears (A); or frequency (total number per ear in brackets) of IFN-γ^+^/TcRβ^+^CD4^+^ T cells following in-vitro re-stimulation of pooled ears with BMDC plus *L. major*-antigen (DC+Ag) (B). (*) or (‡) in 1A indicates a significant difference (0.021<p<0.034) between the number of cells per ear in Healed (*L.m.*-SF) or Healed (SF) versus AMC mice, respectively. (C) Parasite loads in individual ears (circles) or ear DLNs (squares) one week following exposure to infected sand fly bites in AMC and healed animals, (*) p = 0.007.

Healed primary *L. major* infection initiated by needle inoculation of mice has been extensively employed as a model that mimics the clinical practice of leishmanization. Mice with resolved primary lesions harbor *L. major* specific CD4 T cells that simultaneously produce IFN-γ, TNF-α, and IL-2 effector cytokines and mount powerful protective immunity at a site of needle re-challenge, resulting in the rapid control of parasite growth [13],[35]. In order to characterize the protective immune response following natural transmission, 4 *P. duboscqi* sand flies, infected with *L. major* (*L.m.*-SF), were allowed to feed on the ears of C57BL/6 mice with a healed primary lesion in the footpad. Under these conditions, a median of 2 flies will show evidence of blood engorgement, thereby ensuring parasite transmission to a sufficient number of ears to conduct the experiment, while at the same time more faithfully replicating natural transmission, which likely occurs following exposure to a single infected fly. At 1 and 3 days following exposure to the infected flies, a slight but significant increase in infiltrating CD4 T cells was found in the ears of healed mice relative to fly challenged, naïve, age-matched controls (AMC) (**Figure 1A**). At 7 days post-challenge, the number of infiltrating CD4 cells in the healed mice was dramatically increased relative to controls. In order to determine if parasite antigen was required to mediate this recruitment, healed mice were also exposed to uninfected sand fly bites (SF). Both infected or uninfected bites recruited equivalent numbers of T cells at day 3 post-bite, however, parasite antigen appeared necessary for the dramatic increase observed on day 7 (**Figure 1A**). Remarkably, Ag re-stimulation of dermal derived cells revealed *Leishmania*-specific IFN-γ producing CD4+ T cells at the challenge site within 24 hours, a response that gradually increased to 17% of the total CD4 T cell population at 7 days (**Figure 1B**), correlating with a >100 fold reduction in parasite numbers in the skin (**Figure 1C**). Antigen re-stimulation of T cells from the ears of healed mice exposed to uninfected sand fly bites also revealed the presence of *L.m.*-specific IFN-γ producing CD4+ T cells (**Figure 1B**), suggesting that a functional property of these effector cells is their ability to rapidly migrate to sites of tissue inflammation whether antigen is present or not.

### ALM+CpG vaccination fails to protect against infected sand fly challenge

**Figure 2:**
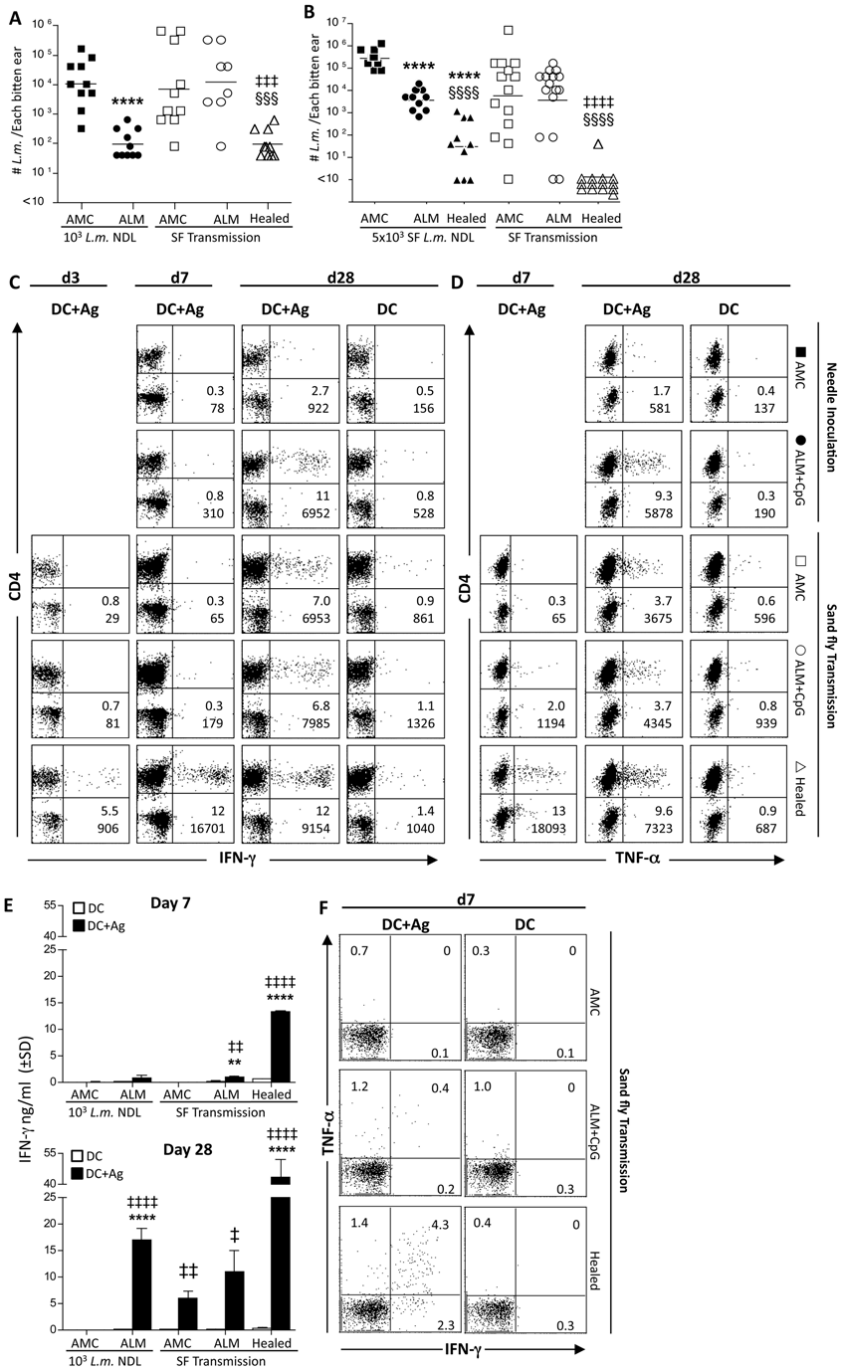
ALM+CpG vaccinated mice are not protected against infected sand fly challenge. Ears of AMC, ALM+CpG vaccinated (ALM), or healed mice were exposed to the bites of 4 *L.m.*-infected sand flies, or needle inoculated with *L.m.* metacyclic promastigotes and subsequently analyzed at the indicated time-points. (A and B) Parasite loads in individual ears 28 days following exposure to infected sand fly bite or needle inoculation with either 10^3^ *L.m.* parasites from culture (A) or 5×10^3^ *L.m.* parasites from infected sand flies (B). (****) p<0.0001 relative to AMC Needle inoculated; (‡‡‡) p = 0.0002, (‡‡‡‡) p<0.0001 relative to AMC sand fly inoculated; (§§§) p = 0.0005, (§§§§) p<0.0001 relative to ALM+CpG. (C and D) Intracellular staining for the frequency (top number) and total number per ear (bottom number) of IFN-γ^+^ (C) or TNF-α^+^ (D) CD4^+^TcRβ^+^ T cells after in-vitro re-stimulation of pooled ear-derived cells from the same groups of mice employed in 2A, with BMDC alone (DC) or DC+Ag. (E) Detection of IFN-γ by ELISA following in-vitro re-stimulation of ear derived cells with DC or DC+Ag. (‡) p = 0.010; (‡‡) 0.003<p<0.004; (‡‡‡‡) p<0.0001 relative to DC; (**) p = 0.004; (****) p<0.0001 relative to AMC DC+Ag. (F) Frequency of TNF-α^+^ and/or IFN-γ^+^ cells among CD3^+^CD4^+^-gated ear derived T cells from the indicated groups following re-stimulation with DC or DC+Ag.

Vaccination with autoclaved *L. major* (ALM), or a recombinant leishmania protein, plus CpG oligodeoxynucleotides (ODN) has been shown to effectively protect against needle challenge with *L. major* in mice [11],[13]. We therefore employed ALM+CpG to test the efficacy of a non-living vaccine against natural transmission. Mice vaccinated with ALM+CpG three times s.c. in the footpad at two week intervals, along with age-matched naïve controls and mice with healed primary lesions, were exposed to the bites of 4 infected sand flies twelve weeks following the last vaccine injection. Four weeks following infected sand fly exposure or needle inoculation, coincident with the time of peak parasitic load in naïve mice, parasite burden in the ear dermis was assessed. Mice with healed primary lesions again dramatically controlled parasite growth following exposure to the bites of infected sand flies (**Figure 2A**). In contrast, ALM+CpG vaccination conferred no protection against transmission by sand fly bite, despite conferring strong protection against needle inoculation. Ear lesion measurements obtained 4 weeks after infection also revealed a compromised benefit of the ALM+CpG vaccine against sand fly challenge (**Figure S1**). Note that despite the comparable parasitic loads in naïve mice following sand fly or needle challenge, the pathology associated with transmission by bite was far more severe.

The respective doses of the fly versus needle inocula did not appear to be a factor in the different outcomes of infection in the ALM+CpG vaccine as naive mice infected via needle or sand fly bite contained similar numbers of parasites in the challenge sites at 4 wks post-infection. In order to address the issue of dose more directly, and to determine if sand fly-derived parasites might be more virulent than those obtained from culture, ALM+CpG vaccinated and healed mice were challenged by infected sand fly bite or by inoculation with a five-fold higher dose of metacyclic promastigotes purified from the midguts of sand flies harboring 14 d, mature infections. Based on previous observations [36], 5×10^3^ sand fly derived parasites are within the projected upper range of the variable doses transmitted following exposure to 4 infected sand flies. Mice with healed primary lesions were again powerfully protected against both needle and sand fly challenge (**Figure 2B**), and the ALM+CpG vaccinated mice maintained their immunity against the higher dose, sand fly-derived, needle inoculum, demonstrating a 100-fold decrease in parasite load. Importantly, these mice again failed to demonstrate any protection against sand fly transmitted infection as measured by either parasite load (**Figure 2B**), or a significant reduction in lesion scores (**Figure S2**). These results strongly suggest that transmission of *L. major* by sand fly bite, rather than an inherent difference in the dose or virulence of sand fly derived parasites, is responsible for the inability of ALM+CpG vaccinated mice to protect against natural challenge.

Kinetic analysis of the immune response among groups of mice challenged by the bite of infected sand flies in Figure 2A revealed that healed mice mounted a rapid and robust *L.m.*-specific response, while ALM+CpG vaccinated mice mounted a much weaker response, as determined by intracellular staining of dermal- derived CD3^+^ T cells for IFN-γ (**Figure 2C**) or TNF-α (**Figure 2D**). These different effector cell frequencies were reflected in the levels of IFN-γ secreted by ear-derived cells, as detected by ELISA (**Figure 2E**). Previous observations suggest that CD4+ T cells capable of producing multiple cytokines in response to antigen stimulation are more effective at protecting against disease [13]. In agreement with these studies, we found that a large proportion of *L.m.*-specific T cells in the healed mice produced IFN-γ and TNF-α simultaneously at day 7 (**Figure 2F**). These results emphasize the correlation between an early response and parasite clearance following sand fly transmission, and explain why ALM+CpG vaccinated mice were unable to control sand fly transmitted infection as compared to healed mice.

We were also interested to understand why the delayed appearance of the Th1 effector response in ALM+CpG vaccinated mice was sufficient to protect against needle challenge but not sand fly challenge. At 4 wks post-infection, despite enhanced numbers of lymphocytes (**Figure S3**) and increased levels of parasite antigen (**Figure 2A**) at the site of infected sand fly bite versus needle inoculation in ALM+CpG vaccinated mice, we observed a decrease in the frequency of both IFN-γ^+^ (6.8% versus 11%) and TNF-α^+^ (3.7% versus 9.3%) *L.m.*-specific T cells (**Figure 2, C and D**), as well as reduced levels of secreted IFN-γ (**Figure 2E**). Thus, conditions in the bite site appear to compromise the activation and/or effector function of the memory response generated by the killed vaccine.

### Sand fly inoculation maintains a localized neutrophilic response

**Figure 3:**
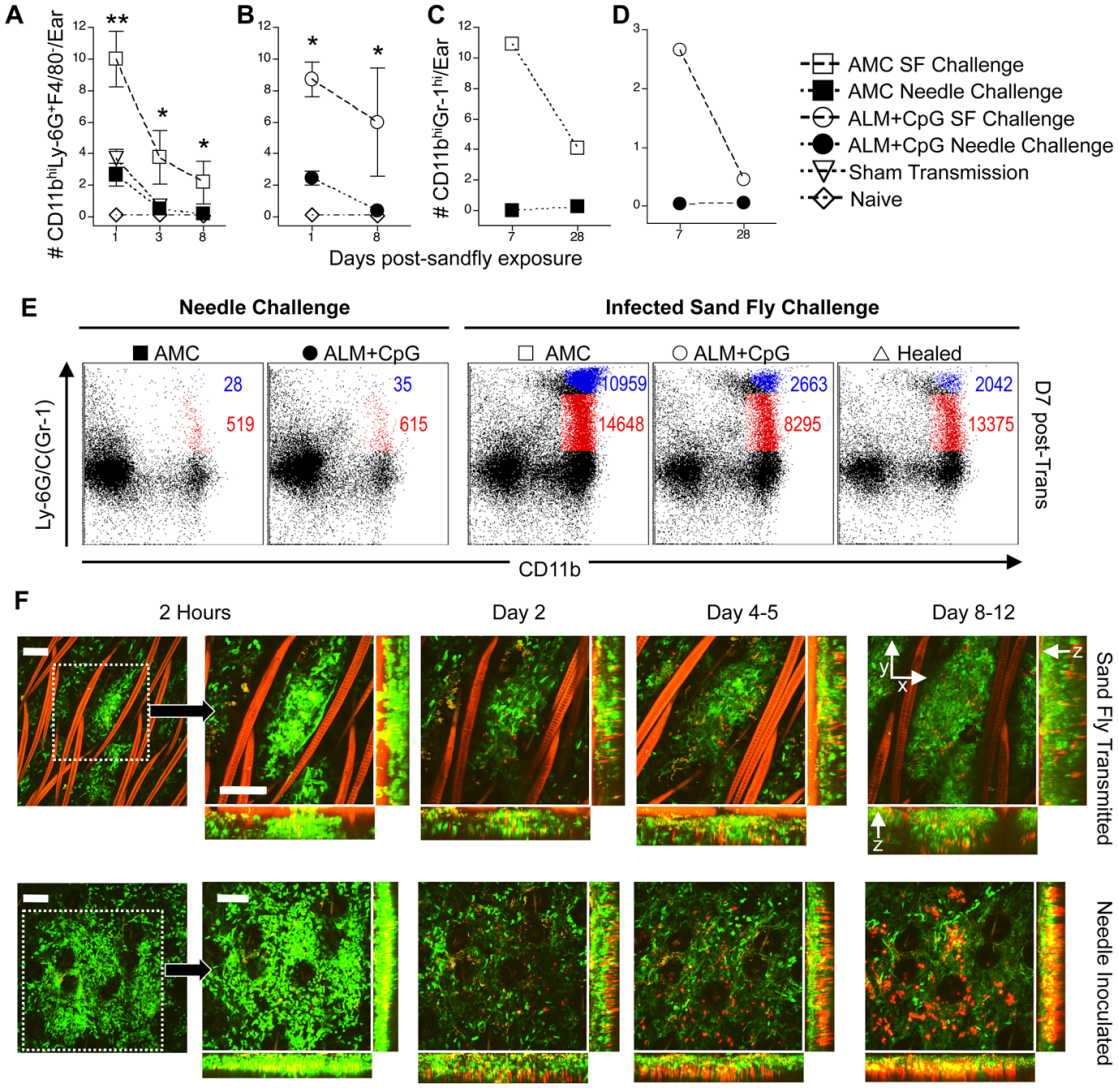
Sand fly bite induces persistent inflammation characterized by maintenance of neutrophils at the dermal bite site. Ears of AMC, ALM+CpG-vaccinated, and healed mice were exposed to the bites of *L.m.*-infected sand flies, needle inoculated with 10^3^ *L.m.*, or manipulated by exposure to empty vials used for sand fly feeding. (A–D) Analysis of the total number of CD11b^+^Ly6G^+^F4/80^−^ neutrophils (A and B) per ear, ±SE (*n* = 4–6 individual ears per group per day) or CD11b^+^Ly-6C/G(Gr-1)^hi^ neutrophils (C and D) per ear (pooled sample) at the indicated times following exposure to 4 *L.m.*-infected sand flies or needle inoculation with 10^3^ sand fly (A–B) or culture (C–D) derived *L.m.*. CD11b^+^Ly-6C/G(Gr-1)^hi^ cells in (C) and (D) are 90–95% MHC II and CD11c negative, and are indicated in blue in 1E. CD11b^+^Ly-6C/G(Gr-1)^int^ cells are 70–75% MHC II positive, 10% CD11c positive, and are indicated in red in 1E. (**) p = 0.001 and (*) 0.016<p<0.036 versus needle inoculated. (E) Representative dot plots of CD11b and Ly-6C/G(Gr-1) expressing cells 7 days following needle inoculation or exposure to the bites of infected sand flies. (F) Visualization of LYS-eGFP^+^ cells at sites of *L.major*-RFP parasite deposition at the indicated times following needle inoculation or infected sand fly bite employing 2P-IVM. Individual inoculation sites were imaged sequentially by identifying patterns of parasite deposition, hair follicles (Orange), and blood vasculature as previously reported [34]. The left panel of images represents a low magnification view of the infection site with the boxed region indicating the area employed for subsequent images in the time series. Scale bars = 50 µM.

We have recently demonstrated that the early host response to sand fly bites is associated with a unique and prolonged recruitment of neutrophils into the localized bite site, resulting in the formation of a “neutrophil plug”, and that the presence of neutrophils during the initiation of infection promotes the establishment of sand fly transmitted disease [34]. In order to explore the possibility that the host inflammatory response to sand fly bite is responsible for the failure of ALM+CpG vaccination to protect against sand fly transmitted infection, we first compared the inflammation induced by sand fly versus needle inoculation of *L. major*. When ear dermal cells from naive (**Figure 3A**) and ALM+CpG vaccinated mice (**Figure 3B**) were analyzed for the presence of neutrophils over the first week of infection, both needle and sand fly inoculated ears revealed a significant recruitment at 24 hours, although sand fly bitten ears had significantly greater numbers (p = 0.001). Importantly, only sand fly bitten ears maintained the neutrophilic infiltrate at the inoculation site at 3 and 8 days post-inoculation (**Figure 3, A and B**). Of note, a very transient neutrophilic response was also observed in the ears of the sham-transmitted mice, elicited by manipulation of the ear dermis during exposure to the transmission apparatus. Examination of cells derived from the ears of the needle or sand fly challenged mice shown in Figure 2A, revealed that only sand fly inoculation maintained recruitment of large numbers of neutrophils at the inoculation site at 1 and even 4 wks post-infection (**Figure 3, C and D**), which at least in the case of the naïve mice, was not explained by differences in the parasitic load. Analysis of all CD11b and Ly-6G/C (Gr-1) expressing cells reveals that increased numbers of neutrophils were also associated with large numbers of CD11b+Gr-1^int^ macrophages/monocytes (**Figure 3E**).

In order to visualize neutrophil recruitment and maintenance at individual sites of *L.m.* inoculation over time, we employed 2-photon intra-vital microscopy (2P-IVM) in conjunction with a red fluorescent protein-expressing strain of *L.m* (*L.m.*-RFP) [36], and naïve mice expressing enhanced green fluorescent protein (eGFP) under the control of the endogenous lysozyme M promotor (LYS-eGFP mice) [37]. As previously reported, the GFP^hi^ cells in these mice are neutrophils [34],[37], and accumulate within both needle and sand fly inoculation sites shortly after infection (**Figure 3F**, 2 hours; and **Video S1** **and** **S2**). The sand fly inoculation site is distinguished by an especially tight co-localization of RFP^+^ parasites and GFP^hi^ neutrophils, which form a plug delineating the site of proboscis penetration. (**Figure 3F**, 2 hours; **Video S1** **and** **S3**). While neutrophils were maintained at the site of parasite deposition by sand fly bite (**Figure 3F**, **Video S4**) this co-localization was rapidly lost at the site of needle inoculation, and the majority of neutrophils present in the field of view at later times were within blood vessels (**Figure 3F** **and** **Video S5**).

### Immunity is enhanced in the absence of neutrophils

**Figure 4:**
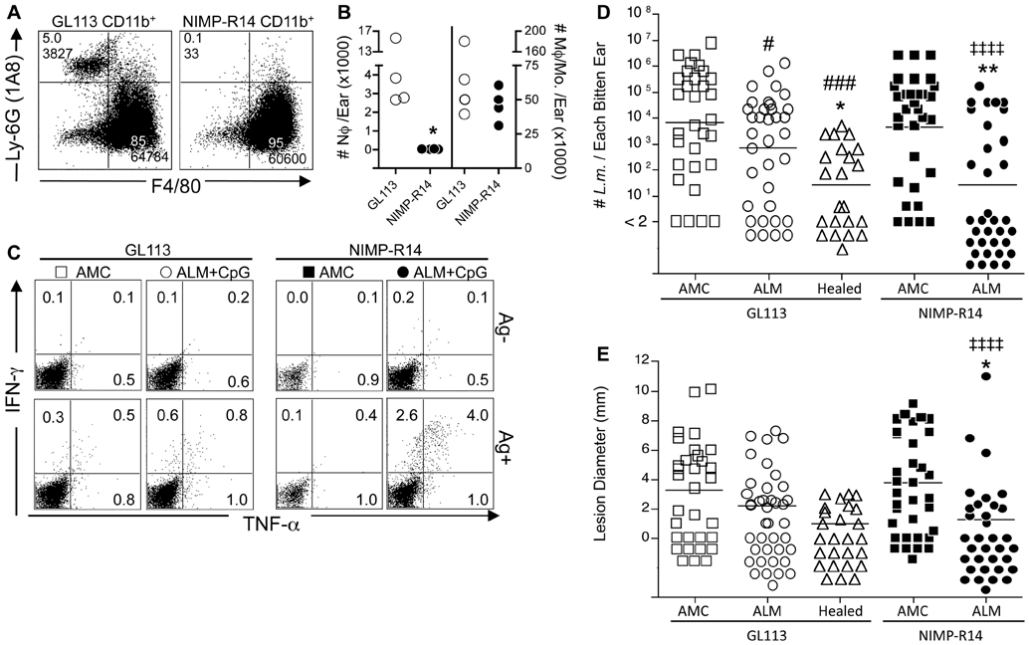
Depletion of neutrophils following sand fly bite enhances the efficacy of ALM+CpG vaccination. AMC, ALM+CpG vaccinated, or healed mice were exposed to the bites of 4 *L.m.*-infected sand flies and subsequently treated with GL113 control IgG (open symbols) or NIMP-R14 neutrophil-depleting Ab (closed symbols) at 3.5, 9 and 14 days following sand fly bite. (A and B) Representative dot plot of CD11b gated, Ly6-G and F4/80 expressing ear cells (A) and the total number of CD11b^+^Ly-6G^+^F4/80^−^ neutrophils and CD11b^+^Ly6G^−^F4/80^+^ macrophage/monocytes per ear (n = 4) (B), among ALM+CpG vaccinated mice 2.5 days following Ab treatment and 6 days following exposure to infected sand flies. (*) p = 0.03 versus GL113. (C) Frequency of TNF-α^+^ and/or IFN-γ^+^ cells among CD3^+^CD4^+^-gated T cells after DC or DC+Ag re-stimulation of ear derived cells 15 days following exposure to infected sand fly bites. Analysis of parasite numbers per ear (D) and lesion diameter (E) among the indicated groups 28 days following exposure to infected sand fly bites. Each data point represents an individual ear in three pooled experiments in which animals from AMC and ALM+CpG groups were treated with GL113 or NIMP-R14 mAb. In 4D: (‡‡‡‡) p<0.0001 versus AMC(NIMP-R14); (**) p = 0.002, (*) p = 0.044 versus ALM+CpG(GL113); (#) p = 0.009, (###) p = 0.0004 versus AMC(GL113). In 4E (‡‡‡‡) p<0.0001 versus AMC(NIMP-R14); (*) p = 0.011 versus ALM+CpG(GL113).

We explored the possibility that neutrophil depletion might rescue the ability of the killed vaccine to confer protection against sand fly transmitted infection. As neutrophils are important for the early establishment of sand fly transmitted infections, their depletion at the time of challenge would, as previously shown [34], promote early resistance and compromise infection even in the naïve mice. Thus, the mice were left untreated for the first 3.5 days following sand fly transmission, then treated on days 3.5, 9, and 14, with a neutrophil depleting Ab [38],[39] or control IgG to mimic the loss of neutrophils observed following needle inoculation, but not sand fly transmission. Analysis of CD11b^+^Ly-6G^+^F4/80^−^ neutrophils and CD11b^+^Ly-6G^−^F4/80^+^ macrophages/monocytes at the site of infection 6 days post-transmission revealed that the neutrophil depletion was both specific and efficient (**Figure 4, A and B**). At 2 weeks post-transmission, the neutrophil depletion promoted stronger Ag-specific IFN-γ and TNF-α responses in the ALM+CpG vaccinated mice (**Figure 4C**). More importantly, the neutrophil depletion enhanced the efficacy of the killed vaccine. Analysis of extensive data pooled from three independent experiments revealed that on day 28 post-transmission, the neutrophil depleted, ALM+CpG-vaccinated mice showed a highly significant reduction in parasite load compared with neutrophil depleted, naïve mice (p<0.0001), as well as control treated, ALM+CpG vaccinated mice (p = 0.002), and indistinguishable from that in healed animals (**Figure 4D**). The enhanced parasite clearance in neutrophil depleted, ALM+CpG vaccinated mice was associated with a significant reduction in lesion size compared with neutrophil depleted, naïve mice (p<0.0001) and control treated, ALM+CpG vaccinated mice (p = 0.01) (**Figure 4E**). Importantly, the neutrophil-depleted, naïve controls did not exhibit lower parasite loads compared with their control treated counterparts, suggesting the effect of neutrophil depletion after the initial establishment of infection, and during the extended period of neutrophil recruitment following transmission by bite, was specific to the vaccine setting.

## Discussion

The generation of a safe, non-living, prophylactic vaccine against leishmaniasis has been largely unsuccessful, a failure that is not explained by the lack of available target antigens with the potential to confer a protective response [40]. Failed human trials reported in the 1990s employing ALM+BCG were particularly perplexing as the same or a similar vaccine has been shown to work well as immunotherapy to hasten cure in patients with active disease [41],[42]. Furthermore, it elicits detectable parasite-specific IFN-γ production and leishmanin skin-test conversion in at least a proportion of recipients [20],[21],[22],[23],[24],[25],[26],[27],[28], and similar vaccine formulations, including the ALM+CpG vaccine employed in this study, have been shown to be highly effective against needle challenge in mouse models [11],[13]. The results reported here suggest that the killed vaccines failed in people because, while generating some correlates of immunity that may provide adequate defense against a needle inoculum, failed to generate and/or maintain the rapid, robust response at the site of secondary challenge induced by leishmanization that is required to prevent disease following delivery of parasites by sand fly bite. The protective response in healed mice is likely associated with the speed with which effector cells appear at a site of tissue damage, irrespective of the presence of parasites (see Figure 1). Following encounter with antigen in the inoculation site, these cells might then provide an immediate burst of effector cytokines, and counteract early on the down-modulatory environment created by the highly localized, neutrophil-dominated, response to sand fly bite. In contrast, and despite the ability of the killed vaccine plus CpG to generate multi-functional, effector T cells protective against needle challenge [13] (see also Figure 4), these cells are not present in adequate numbers and at sufficiently early time points to protect against sand fly transmission. This point is emphasized by the presence of similar numbers of neutrophils in both ALM+CpG vaccinated and healed mice one week following exposure to infected sand flies (Figure 3E), yet only healed mice were protected. Both the rapidity of the effector response in healed mice, as well as the fact that these cells were recruited by uninfected sand fly bites, suggests that these cells are not derived from a “central” memory population, that would require antigen encounter and several rounds of division in the DLN before gaining effector function [43]. More likely, the rapid appearance of these cells in the challenge site reflects a pre-existent, tissue-seeking effector population, undergoing constant renewal by the persistence of viable organisms in the healed mice [3],[4]. Further understanding of the effector population maintained by persistent infection, including the role of CD8+ cells, is likely to be highly informative to strategies of successful vaccination [44].

A critical question remains the mechanism by which neutrophil persistence following sand fly transmission inhibits parasite elimination in ALM+CpG vaccinated mice. Phagocyte clearance of apoptotic neutrophils during the resolution of inflammation has a known inhibitory effect on macrophage functions [45], and DC functions are similarly impaired following uptake of apoptotic neutrophils [46]. Thus, infected macrophages and DC persistently exposed to apoptotic neutrophils at the site of sand fly bite are likely to be refractory to activation signals, inhibiting both the killing and APC functions of these cells. This is especially relevant to sand fly transmission where the association between neutrophils and macrophage/monocytes, as well as dendritic cells, is highly localized at the sand fly bite site. Apoptosis of infected neutrophils has been readily captured by 2P-IVM [34]. The maintenance of neutrophils at sand fly bite sites is likely the result of conditions leading to their protracted recruitment, as opposed to prolongation of their life span in the skin [47]. Thus, while the initial recruitment of neutrophils may be driven primarily by tissue injury, their continued presence is likely influenced by PSG or salivary components, that are themselves chemotactic or that initiate the inflammatory cascade [33].

The results reported here represent the first determination, so far as we are aware, of the factors influencing the efficacy of protective immunity generated by different vaccine formulations against sand fly challenge, and may be relevant to the conditions that modulate vaccine induced immunity to other vector borne pathogens. Beyond emphasizing the somewhat obvious importance of using natural challenge models to evaluate experimental vaccines against leishmaniasis, the results provide a more stringent set of screening criteria that might be used to predict vaccine success against fly challenge, relating to the rapid appearance of multifunctional effector cells within the challenge site.

Pertinent to our findings are those of Rogers et. al. who demonstrated that vaccination of BALB/c with glycoconjugates derived from PSG diminishes disease severity following sand fly challenge [29]. Collectively, these findings should be especially informative for ongoing and future clinical development of “second-generation” *Leishmania* vaccines [48], and reinforce the rationale for inclusion of molecules specific to natural transmission, such as selected components of sand fly saliva or promastigote-secretory gel, in an anti-Leishmania vaccine [49].

## Materials and Methods

### Mice

Female C57BL/6 mice were obtained from Jackson Laboratories. C57BL/6 LYS-eGFP knock-in mice [37] were a gift from T. Graf (Albert Einstein University, NY) and were bred at Taconic Laboratories through a contract with the NIAID. Mice were maintained at a NIAID animal care facility under specific pathogen-free conditions. All animal experiments were performed under a study protocol approved by the NIAID Animal Care and Use Committee.

### *Leishmania* cell lines

All experiments were carried out using the *L. major* Friedlin strain obtained from the Jordan Valley NIH/FV1 (MHOM/IL/80/Friedlin). In some experiments, a stable transfected line of FV1 *L. major* promastigotes expressing a red fluorescent protein was employed, as described previously [36]. Briefly, the DsRed gene was amplified by PCR employing the pCMV-DsRed-Express plasmid (BD Biosciences/Clontech) as a template and cloned into the SpeI site of the pKSNEO Leishmania expression plasmid. FV1 promastigotes were transfected with the resulting expression plasmid construct [pKSNEO-DsRed] and selected for growth in the presence of 50 µg/ml Geneticin (G418) (Sigma).

### Parasite preparation for needle inoculation

*L. major* or *L. major*-RFP were grown at 26°C in medium 199 supplemented with 20% heat-inactivated FCS (Gemini Bio-Products), 100 U/ml penicillin, 100 µg/ml streptomycin, 2 mM L-glutamine, 40 mM Hepes, 0.1 mM adenine (in 50 mM Hepes), 5 mg/ml hemin (in 50% triethanolamine), and 1 mg/ml 6-biotin. Infective-stage metacyclic promastigotes were isolated from stationary cultures (4–6 day-old) by negative selection of non-infective forms using peanut agglutinin [50] (PNA, Vector Laboratories Inc). In some experiments metacyclic promastigotes of *L. major* were isolated from sand flies on day 14 following infection with *L. major*, as previously described [36]. Briefly, Infected flies were killed, dissected aseptically, and the stomodeal valve and anterior gut of each fly was transferred into Dulbecco's modified Eagle's medium (DMEM). The guts were macerated briefly using a plastic pestle, spun twice to remove the debris, and washed once in DMEM followed by metacyclic promastigote isolation as described above. Mice were subsequently infected with the specified number of parasites in the ear dermis by intra-dermal (i.d.) injection using a 29 ½ GA needle in a volume of 10 µl unless specified otherwise.

### Healed mice and Autoclaved *Leishmania* antigen (ALM) plus CpG oligodeoxynucleotides (ODN) vaccination

Analysis of protective immunity in mice with a healed primary lesion was carried out using animals that had been infected 16–20 weeks previously with 10^4^ *L. major* metacyclic promastigotes in the left hind footpad by sub-cutaneous injection using a 29 ½ gauge needle in a volume of 40 µl. Autoclaved *Leishmania* antigen (ALM) plus CpG oligodeoxynucleotides (ODN) vaccination was performed in a manner similar to that published previously [11]. Briefly, B6 mice were injected subcutaneously in their left hind footpad with 50 mg of clinical grade ALM, prepared from whole cell heat-killed *L. major* promastigotes (WHO) plus 50 µg of CpG ODN sequence 1826 (Coley Pharmaceutical Group), graciously provided by Dr. P. Darrah (VRC/NIH), using a 29 ½ gauge needle in a volume of 40 µl, three times, at 2 week intervals.

### Infection of sand flies and transmission of *L. major* to mice

Transmission of *L. major* parasites was performed as described [34],[36]. Briefly, 2–4 day old *P. duboscqi* (Mali colony) female sand flies were infected via feeding through a chick skin membrane on heparinized mouse blood containing *L. major* or *L. major*-RFP amastigotes or promastigotes. After 14–15 days, individual flies were transferred to plastic vials covered at one end with nylon mesh. Mice were anesthetized by intraperitoneal injection of 30 µl of ketamine/rompin (100 mg/ml). Specially designed clamps were used to bring the mesh end of each vial into contact with the ear of an anesthetized mouse, allowing flies inside the vial to feed on the ear skin for a period of 2 to 3 hours in the dark. In some experiments mice were exposed to empty vials. The number of flies with blood meals was employed as a means of checking for equivalent exposure to potential transmission by sand fly bite among animals in different treatment groups. The median number of flies with blood meals in vials with 4 flies was 2.

### Processing of tissue

Ear tissue was prepared as previously described [34]. Briefly, the ventral and dorsal sheets of needle or sand fly inoculated ears were separated, deposited in DMEM containing 100 U/ml penicillin, 100 µg/ml streptomycin and 0.2 mg/ml Liberase CI purified enzyme blend (Roche Diagnostic Corp.), and incubated for 2 hours at 37°C and 5% CO_2_. Digested ear sheets were subsequently homogenized for 3 minutes using the Medicon/Medimachine tissue homogenizer system (Beckton Dickinson). Individual retromaxillary (ear) lymph nodes were removed, and mechanically dissociated using tweezers and a syringe plunger. Single cell suspensions of tissue homogenates were then filtered using a 70 µm-pore size Falcon cell strainer (BD Biosciences).

### Phenotypic analysis of ear derived cell populations

Mice were sacrificed and single cell suspensions from the ear dermis were obtained as described above. Cells were incubated without fixation with an anti-Fc-γ III/II (CD16/32) receptor Ab (2.4G2, BD Biosciences) in RPMI without phenol red (Gibco) containing 1.0% FCS for 10” followed by incubation for 20” with a combination of 4 or 6 of the following anti-mouse antibodies: PE-Cy7 or APC anti-CD11b (M1/70 BD Biosciences); Per-CP Cy5.5 anti-Gr-1(Ly6G/C) (RB6-8C5, BD Biosciences); FITC or PE anti-Ly6G (1A8, BD Biosciences); PE anti-CD11c (HL3, BD Biosciences); Per-CP Cy5.5 anti-CD11c (N418, BioLegend); APC anti-F4/80 (BM8, eBioscience), FITC anti-I-A^b^ (AF6-120.1, BD Biosciences); or Alexafluor-700 anti-mouse MHC II (M5/114.15.2, eBioscience). The isotype controls employed were rat IgG1 (R3-34) and rat IgG2b (A95-1). The data were collected and analyzed using CELLQuest software and a FACScalibur or FacsDIVA software and a FacsCANTO flow cytometer (BD Biosciences). Gating of ear-derived cells was carried out as described previously [34]. Ears were analyzed individually, or pooled with ears from the same group, as indicated in the text.

### Restimulation of ear derived T cells for cytokine analysis by flow cytometry or ELISA

Whole ear single-cell suspensions in RPMI 1640 containing 10% FCS, 10 mM Hepes, L-glutamine, and penicillin/streptomycin, obtained as described above, were incubated at 37°C in 5% CO_2_ for 16–18 hours in flat-bottom 48-well plates with 2.5×10^5^ BMDCs, with or without 50 mg/ml freeze-thaw *Leishmania* antigen prepared from *L. major* V1 stationary phase promastigotes, in a final volume of 1 ml. During the last 5–6 hours of culture Brefeldin A (Golgiplug; BD Biosciences) was added to block golgi transport according the manufacturers' instructions. Following in vitro culture, cells were washed and stained with anti-Fc III/II (CD16/32) receptor Ab (2.4G2) for 10 minutes in RPMI without phenol red containing 1.0% FCS, followed by PE-Cy7 or PE-Cy5 anti-mouse CD4 (RM4-5) for 15 minutes. In some experiments cells were also stained with FITC anti-TcR β (145-2 C11). Cells were then fixed with BD Cytofix/Cytoperm (BD Biosciences) and stained with anti-Fc III/II (CD16/32) receptor Ab (2.4G2) followed by a combination of the following anti-mouse antibodies: PerCP-Cy5.5 anti-CD3 (145-2C11), FITC-, APC-, or AlexaFluor 700 anti-IFN-g (XMG1.2), and FITC or PE anti-TNF-α (MP6-XT22). Intracellular staining was carried out for 30 minutes on ice. All antibodies were acquired from BD Biosciences. For each sample, greater then or equal to 4000 CD4^+^CD3^+^ cells were collected using a FACS Caliber or FACS Canto flow cytometer and analyzed using either Cell Quest Pro or FACS Diva Software, respectively (BD Biosciences). For measurement of IFN-γ in culture supernatants, pooled, single-cell suspensions of ear tissue as described above were incubated in triplicate at 37°C in 5% CO_2_ for 72 hours in 96-well round bottom plates with 2.5×10^5^/ml BMDC with or without freeze-thaw *Leishmania* antigen in a total volume of 200 ml. Following incubation, the concentration of IFN-γ in the culture supernatant was determined by ELISA according the manufactures instructions (eBioscience).

### Estimation of parasite load and determination of lesion size

Parasite titrations were performed as previously described [31]. Briefly, tissue homogenates were serially diluted in 96-well flat-bottom microtiter plates containing biphasic medium, prepared using 50 µl NNN medium containing 20% of defibrinated rabbit blood and overlaid with 100 µl M199/S. The number of viable parasites in each ear was determined from the highest dilution at which promastigotes could be grown out after 7–10 days of incubation at 26°C.

Because individual sand flies, or more then one sand fly may deposit parasites in more than one location, sand fly bitten ears often have more then one lesion. Total lesion diameter was determined by measuring the diameter of individual lesions using a caliper and in cases where there was more then one lesion per ear the diameters were added together.

### Two photon intravital skin imaging and image analysis

Two photon intravital imaging and image analysis was performed as described previously [34]. Briefly, anesthetized mice were imaged in the lateral recumbent position that allowed the ventral side of the ear pinna to rest on a coverslip. A strip of Durapore tape (3 M) was stuck to a bench top several times (to ensure that subsequent removal would not cause undue damage) and placed lightly over the ear pinna and affixed to the imaging platform in order to immobilize the tissue. Care was taken to minimize pressure on the ear.

Images were acquired using an inverted LSM 510 NLO multiphoton microscope (Carl Zeiss Microimaging) enclosed in an environmental chamber that was maintained at 30°C. This system had been custom fitted with 3 external non-descanned PMT detectors in the reflected light path. Images were acquired using either a 20×/0.8 air objective or a 25×/0.8 NA water immersion objective. Fluorescence excitation was provided by a Chamelon XR Ti:Sapphire laser (Coherent) tuned to 920 nm for eGFP excitation. Voxel dimensions were 0.64×0.64×2 µm using the 20× objective and 0.36–0.51×0.36–0.51×2 µm using the 25× objective.

Raw imaging data were processed with Imaris (Biplane) using a Gaussian filter for noise reduction. All images are displayed as 2D maximum intensity projections. Movie files of 3-dimentional images were generated using Imaris.

### Neutrophil depletion

Animals were treated with three 0.5 mg injections of a neutrophil depleting (NIMP-R14) or control (GL113) IgG antibody, i.p., on days 3.5, 9, and 14 following sand fly transmission. The first dose of antibody was delayed until 3.5 days after exposure to infected sand fly bite as earlier observations demonstrated that *L.m.* infection is established in macrophages at this time [34]. Antibody treatments were spaced 5 days apart as preliminary experiments suggested excessive administration of the NIMP-R14 antibody, such as on successive days, led to depletion of cell types other then neutrophils. Success and specificity of depletions were determined as described in the text. The NIMP-R14 hybridoma was a gift from Dr. Y. Belkaid (NIAID).

### Statistical analysis

Parasite loads in the ears of mice transmitted with *L. major* by infected sand fly bite do not follow a Gaussian distribution. This is likely the result of variability in the infectious burden and feeding behavior of individual, infected, sand flies [36]. Therefore, data sets were compared using a nonparametric Mann Whitney test. Mann Whitney calculations were done using Prism 4 (Graphpad Software, Inc. San Diego, CA). In Figure 4, D and E, parasite loads and lesion size were compared using an exact stratified Wilcoxon rank sum test, stratified by experiment in order to allow pooling of experiments as described in the text. The stratified Wilcoxon calculations were done in StatXact 8 Procs (Cytel, Inc., Cambridge, MA). Comparisons in which the data represented replicate samples were carried out using t-tests. All p-values are two-sided.

## Supporting Information

Figure S1 ALM+CpG vaccination reduces lesion size following needle, but not infected sand fly, challenge. Ears of AMC, ALM+CpG vaccinated (ALM), or healed mice were exposed to the bites of 4 *L.m.*-infected sand flies, or needle inoculated with 10^3^ *L.m.* metacyclic promastigotes. Four weeks later, the cumulative lesion diameter per ear was determined as described in Materials and Methods. (*) p = 0.04 versus AMC needle inoculated; (‡) p = 0.009 versus AMC sand fly inoculated. Lesion scores are from those mice depicted in Figure 2A.
(0.37 MB TIF)

Figure S2 ALM+CpG vaccination reduces lesion size following needle challenge with 5×10^3^ sand fly derived *L.m.* metacyclic promastigotes, but not following exposure to infected sand fly challenge. Ears of AMC, ALM+CpG vaccinated (ALM), or healed mice were exposed to the bites of 4 *L.m.*-infected sand flies, or needle inoculated with 5×10^3^ sand fly derived *L.m.* metacyclic promastigotes. At 4 weeks post-challenge, the cumulative lesion diameter per ear was determined as described in materials and methods. (*) p = 0.006, (****) p<0.0001 versus AMC needle inoculated; (‡) p = 0.037 versus AMC sand fly inoculated. Lesions scores are from those ears depicted in Figure 2B.
(0.39 MB TIF)

Figure S3 Kinetic analysis of lymphocyte recruitment to sites of needle or sand fly inoculation of *L. major*. Single cell suspensions of individual ears (n = 8–10) from the groups depicted in Figure 2A and Figure 3C–E, following exposure to the bites of 4 *L.m.*-infected sand flies (white square, Age Matched Control (AMC); white circle, Autoclaved *Leishmania major* (ALM)+CpG; white triangle, Healed) or needle inoculated with 10^3^ *L.m.* metacyclic promastigotes (black square, AMC; black circle, ALM+CpG) were pooled, mixed 1∶1 with trypan blue, and the number of live lymphocytes per ear was determined by trypan blue exclusion and morphology.
(0.45 MB TIF)

Video S1 Formation of neutrophil “plugs” and *L. major* deposition at acute time points following infected sand fly bite. Two-dimentional (XY) image series through the Z-plane from a LYS-eGFP mouse 2 hours following exposure of the ventral ear pinna to the bites of *L.m.*-RFP infected sand flies. Movie is derived from the 2 hour infected sand fly bite image depicted in Figure 3F. The first slice is the ventral ear surface. Playback speed is 4 Z-slices per second. Dimensions of the imaging field are 369(Y)×369(Y)×76(Z) µm.
(0.89 MB MOV)

Video S2 Neutrophil recruitment to a site of parasite deposition at acute time points following needle inoculation of *L. major*. Two-dimentional (XY) image series through the Z-plane from a LYS-eGFP mouse 2 hours following intra-dermal needle inoculation of the ventral ear pinna with 500 *L.m.*-RFP metacyclic promastigotes in 2 µl. Movie is derived from the 2 hour needle inoculated image depicted in Figure 3F. The first slice is the ventral ear surface. Playback speed is 4 Z-slices per second. Dimensions of the imaging field are 521(Y)×521(Y)×82 (Z) µm.
(1.19 MB MOV)

Video S3 Formation of neutrophil “plugs” and *L. major* deposition following infected sand fly bite. Second example of a 2-dimentional (XY) image series through the Z-plane from a LYS-eGFP mouse 2 hours following exposure of the ventral ear pinna to the bites of *L.m.*-RFP infected sand flies. The first slice is the ventral ear surface. Playback speed is 2 Z-slices per second. Dimensions of the imaging field are 280(X)×369(Y)×88(Z) µm.
(0.69 MB MOV)

Video S4 Neutrophil recruitment to a site of parasite deposition by sand fly inoculation. Two-dimentional (XY) image series through the Z-plane from a LYS-eGFP mouse 8 days following exposure of the ventral ear pinna to the bites of *L.m.*-RFP infected sand flies. Movie is derived from the day 8–12 infected sand fly bite image depicted in Figure 3F. The first slice is the ventral ear surface. Playback speed is 4 Z-slices per second. Dimensions of the imaging field are 369(Y)×369(Y)×76(Z) µm.
(0.72 MB MOV)

Video S5 Neutrophil recruitment to a site of parasite deposition by needle inoculation. Two-dimentional (XY) image series through the Z-plane from a LYS-eGFP mouse 12 days following needle inoculation of the ventral ear pinna with 500 *L.m.*-RFP metacyclic promastogites in 2 µl. Movie is derived from the day 8–12 needle inoculated image depicted in Figure 3F. The first slice is the ventral ear surface. Playback speed is 4 Z-slices per second. Dimensions of the imaging field are 521(Y)×521(Y)×82 (Z) µm.
(1.03 MB MOV)

## References

[1] Bates PA (2007). Transmission of Leishmania metacyclic promastigotes by phlebotomine sand flies.. Int J Parasitol.

[2] Schubach A, Haddad F, Oliveira-Neto MP, Degrave W, Pirmez C (1998). Detection of Leishmania DNA by polymerase chain reaction in scars of treated human patients.. J Infect Dis.

[3] Uzonna JE, Wei G, Yurkowski D, Bretscher P (2001). Immune elimination of Leishmania major in mice: implications for immune memory, vaccination, and reactivation disease.. J Immunol.

[4] Belkaid Y, Hoffmann KF, Mendez S, Kamhawi S, Udey MC (2001). The role of interleukin (IL)-10 in the persistence of Leishmania major in the skin after healing and the therapeutic potential of anti-IL-10 receptor antibody for sterile cure.. J Exp Med.

[5] Greenblatt CL (1980). The present and future of vaccination for cutaneous leishmaniasis.. Prog Clin Biol Res.

[6] Kellina OI (1981). Problem and current lines in investigations on the epidemiology of leishmaniasis and its control in the U.S.S.R.. Bull Soc Pathol Exot Filiales.

[7] Nadim A, Javadian E, Tahvildar-Bidruni G, Ghorbani M (1983). Effectiveness of leishmanization in the control of cutaneous leishmaniasis.. Bull Soc Pathol Exot Filiales.

[8] Handman E (2001). Leishmaniasis: current status of vaccine development.. Clin Microbiol Rev.

[9] Palatnik-de-Sousa CB (2008). Vaccines for leishmaniasis in the fore coming 25 years.. Vaccine.

[10] Gurunathan S, Prussin C, Sacks DL, Seder RA (1998). Vaccine requirements for sustained cellular immunity to an intracellular parasitic infection.. Nat Med.

[11] Rhee EG, Mendez S, Shah JA, Wu CY, Kirman JR (2002). Vaccination with heat-killed leishmania antigen or recombinant leishmanial protein and CpG oligodeoxynucleotides induces long-term memory CD4+ and CD8+ T cell responses and protection against leishmania major infection.. J Exp Med.

[12] Coler RN, Skeiky YA, Bernards K, Greeson K, Carter D (2002). Immunization with a polyprotein vaccine consisting of the T-Cell antigens thiol-specific antioxidant, Leishmania major stress-inducible protein 1, and Leishmania elongation initiation factor protects against leishmaniasis.. Infect Immun.

[13] Darrah PA, Patel DT, De Luca PM, Lindsay RW, Davey DF (2007). Multifunctional TH1 cells define a correlate of vaccine-mediated protection against Leishmania major.. Nat Med.

[14] Iborra S, Parody N, Abanades DR, Bonay P, Prates D (2008). Vaccination with the Leishmania major ribosomal proteins plus CpG oligodeoxynucleotides induces protection against experimental cutaneous leishmaniasis in mice.. Microbes Infect.

[15] Palatnik-de-Sousa CB, Barbosa Ade F, Oliveira SM, Nico D, Bernardo RR (2008). FML vaccine against canine visceral leishmaniasis: from second-generation to synthetic vaccine.. Expert Rev Vaccines.

[16] Spath GF, Lye LF, Segawa H, Sacks DL, Turco SJ (2003). Persistence without pathology in phosphoglycan-deficient Leishmania major.. Science.

[17] Uzonna JE, Spath GF, Beverley SM, Scott P (2004). Vaccination with phosphoglycan-deficient Leishmania major protects highly susceptible mice from virulent challenge without inducing a strong Th1 response.. J Immunol.

[18] Mendez S, Gurunathan S, Kamhawi S, Belkaid Y, Moga MA (2001). The potency and durability of DNA- and protein-based vaccines against Leishmania major evaluated using low-dose, intradermal challenge.. J Immunol.

[19] Noazin S, Modabber F, Khamesipour A, Smith PG, Moulton LH (2008). First generation leishmaniasis vaccines: a review of field efficacy trials.. Vaccine.

[20] Antunes CM, Mayrink W, Magalhaes PA, Costa CA, Melo MN (1986). Controlled field trials of a vaccine against New World cutaneous leishmaniasis.. Int J Epidemiol.

[21] Sharifi I, FeKri AR, Aflatonian MR, Khamesipour A, Nadim A (1998). Randomised vaccine trial of single dose of killed Leishmania major plus BCG against anthroponotic cutaneous leishmaniasis in Bam, Iran.. Lancet.

[22] Momeni AZ, Jalayer T, Emamjomeh M, Khamesipour A, Zicker F (1999). A randomised, double-blind, controlled trial of a killed L. major vaccine plus BCG against zoonotic cutaneous leishmaniasis in Iran.. Vaccine.

[23] Khalil EA, El Hassan AM, Zijlstra EE, Mukhtar MM, Ghalib HW (2000). Autoclaved Leishmania major vaccine for prevention of visceral leishmaniasis: a randomised, double-blind, BCG-controlled trial in Sudan.. Lancet.

[24] Armijos RX, Weigel MM, Calvopina M, Hidalgo A, Cevallos W (2004). Safety, immunogenecity, and efficacy of an autoclaved Leishmania amazonensis vaccine plus BCG adjuvant against New World cutaneous leishmaniasis.. Vaccine.

[25] Castes M, Blackwell J, Trujillo D, Formica S, Cabrera M (1994). Immune response in healthy volunteers vaccinated with killed leishmanial promastigotes plus BCG. I: Skin-test reactivity, T-cell proliferation and interferon-gamma production.. Vaccine.

[26] Mahmoodi M, Khamesipour A, Dowlati Y, Rafati S, Momeni AZ (2003). Immune response measured in human volunteers vaccinated with autoclaved Leishmania major vaccine mixed with low dose of BCG.. Clin Exp Immunol.

[27] Bahar K, Dowlati Y, Shidani B, Alimohammadian MH, Khamesipour A (1996). Comparative safety and immunogenicity trial of two killed Leishmania major vaccines with or without BCG in human volunteers.. Clin Dermatol.

[28] Velez ID, Gilchrist K, Arbelaez MP, Rojas CA, Puerta JA (2005). Failure of a killed Leishmania amazonensis vaccine against American cutaneous leishmaniasis in Colombia.. Trans R Soc Trop Med Hyg.

[29] Rogers ME, Sizova OV, Ferguson MA, Nikolaev AV, Bates PA (2006). Synthetic glycovaccine protects against the bite of leishmania-infected sand flies.. J Infect Dis.

[30] Titus RG, Ribeiro JM (1988). Salivary gland lysates from the sand fly Lutzomyia longipalpis enhance Leishmania infectivity.. Science.

[31] Belkaid Y, Kamhawi S, Modi G, Valenzuela J, Noben-Trauth N (1998). Development of a natural model of cutaneous leishmaniasis: powerful effects of vector saliva and saliva preexposure on the long-term outcome of Leishmania major infection in the mouse ear dermis.. J Exp Med.

[32] Rogers ME, Ilg T, Nikolaev AV, Ferguson MA, Bates PA (2004). Transmission of cutaneous leishmaniasis by sand flies is enhanced by regurgitation of fPPG.. Nature.

[33] Teixeira CR, Teixeira MJ, Gomes RB, Santos CS, Andrade BB (2005). Saliva from Lutzomyia longipalpis induces CC chemokine ligand 2/monocyte chemoattractant protein-1 expression and macrophage recruitment.. J Immunol.

[34] Peters NC, Egen JG, Secundino N, Debrabant A, Kimblin N (2008). In vivo imaging reveals an essential role for neutrophils in leishmaniasis transmitted by sand flies.. Science.

[35] Tabbara KS, Peters NC, Afrin F, Mendez S, Bertholet S (2005). Conditions influencing the efficacy of vaccination with live organisms against Leishmania major infection.. Infect Immun.

[36] Kimblin N, Peters N, Debrabant A, Secundino N, Egen J (2008). Quantification of the infectious dose of Leishmania major transmitted to the skin by single sand flies.. Proc Natl Acad Sci U S A.

[37] Faust N, Varas F, Kelly LM, Heck S, Graf T (2000). Insertion of enhanced green fluorescent protein into the lysozyme gene creates mice with green fluorescent granulocytes and macrophages.. Blood.

[38] Tacchini-Cottier F, Zweifel C, Belkaid Y, Mukankundiye C, Vasei M (2000). An immunomodulatory function for neutrophils during the induction of a CD4+ Th2 response in BALB/c mice infected with Leishmania major.. J Immunol.

[39] McFarlane E, Perez C, Charmoy M, Allenbach C, Carter KC (2008). Neutrophils contribute to development of a protective immune response during onset of infection with Leishmania donovani.. Infect Immun.

[40] Launois P, Tacchini-Cottier F, Kieny MP (2008). Cutaneous leishmaniasis: progress towards a vaccine.. Expert Rev Vaccines.

[41] Convit J, Ulrich M, Zerpa O, Borges R, Aranzazu N (2003). Immunotherapy of american cutaneous leishmaniasis in Venezuela during the period 1990–99.. Trans R Soc Trop Med Hyg.

[42] Musa AM, Khalil EA, Mahgoub FA, Elgawi SH, Modabber F (2008). Immunochemotherapy of persistent post-kala-azar dermal leishmaniasis: a novel approach to treatment.. Trans R Soc Trop Med Hyg.

[43] Zaph C, Uzonna J, Beverley SM, Scott P (2004). Central memory T cells mediate long-term immunity to Leishmania major in the absence of persistent parasites.. Nat Med.

[44] Scott P, Artis D, Uzonna J, Zaph C (2004). The development of effector and memory T cells in cutaneous leishmaniasis: the implications for vaccine development.. Immunol Rev.

[45] Savill J, Fadok V (2000). Corpse clearance defines the meaning of cell death.. Nature.

[46] Stuart LM, Lucas M, Simpson C, Lamb J, Savill J (2002). Inhibitory effects of apoptotic cell ingestion upon endotoxin-driven myeloid dendritic cell maturation.. J Immunol.

[47] Gregory CD, Devitt A (2004). The macrophage and the apoptotic cell: an innate immune interaction viewed simplistically?. Immunology.

[48] Coler RN, Reed SG (2005). Second-generation vaccines against leishmaniasis.. Trends Parasitol.

[49] Titus RG, Bishop JV, Mejia JS (2006). The immunomodulatory factors of arthropod saliva and the potential for these factors to serve as vaccine targets to prevent pathogen transmission.. Parasite Immunol.

[50] Sacks DL, Hieny S, Sher A (1985). Identification of cell surface carbohydrate and antigenic changes between noninfective and infective developmental stages of Leishmania major promastigotes.. J Immunol.

