# Supplementary figures and images for: Vector Transmission of Leishmania Abrogates Vaccine-Induced Protective Immunity
Source: PLoS Pathog. 2009 Jun 19;5(6):e1000484. doi: 10.1371/journal.ppat.1000484 (PMC2691580; doi:10.1371/journal.ppat.1000484)

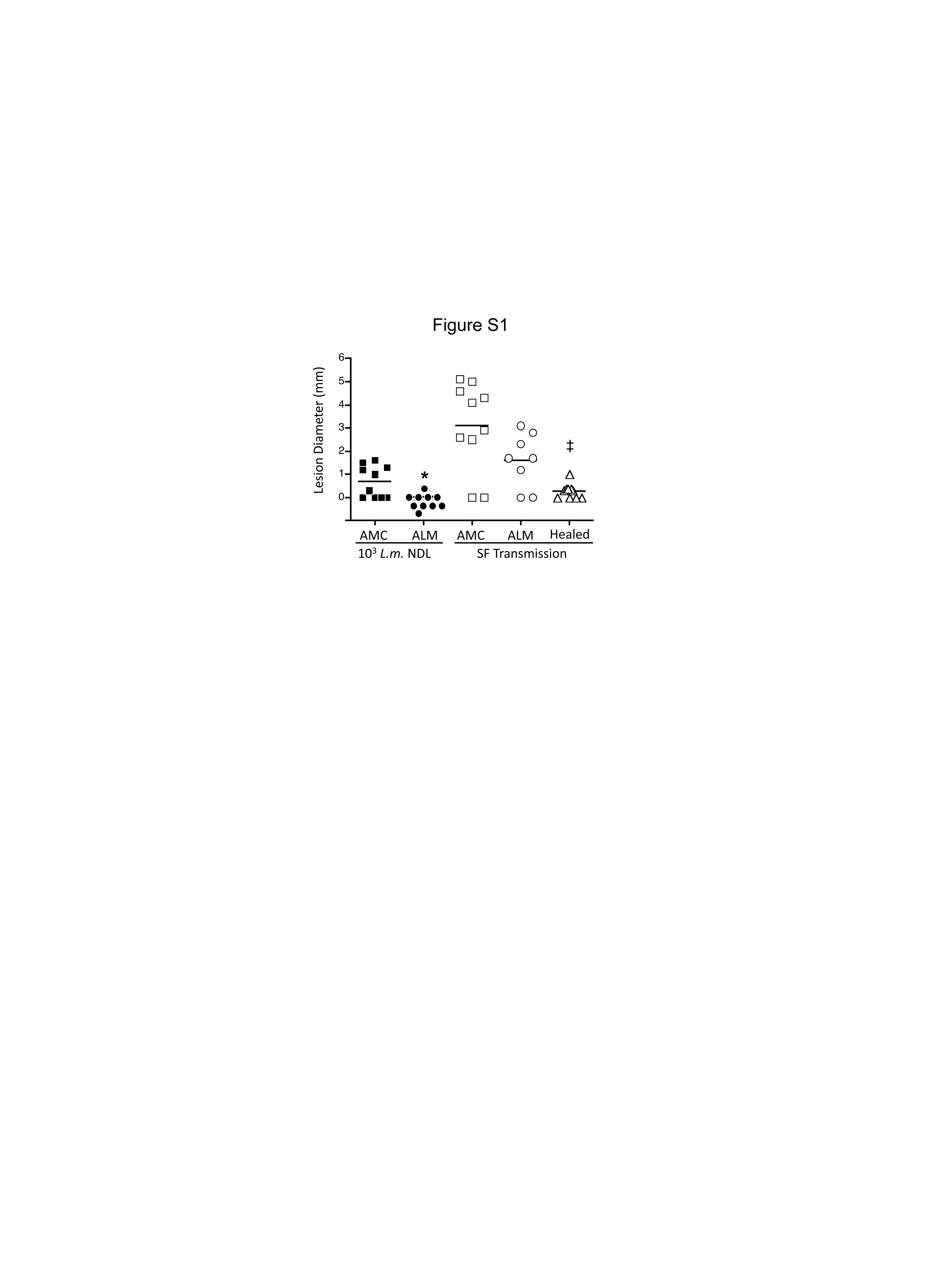

Supplement: Figure S1 — ALM+CpG vaccination reduces lesion size following needle, but not infected sand fly, challenge. Ears of AMC, ALM+CpG vaccinated (ALM), or healed mice were exposed to the bites of 4 L.m.-infected sand flies, or needle inoculated with 103 L.m. metacyclic promastigotes. Four weeks later, the cumulative lesion diameter per ear was determined as described in Materials and Methods. (*) p = 0.04 versus AMC needle inoculated; (‡) p = 0.009 versus AMC sand fly inoculated. Lesion scores are from those mice depicted in Figure 2A. (0.37 MB TIF) [file ppat.1000484.s001.tif]

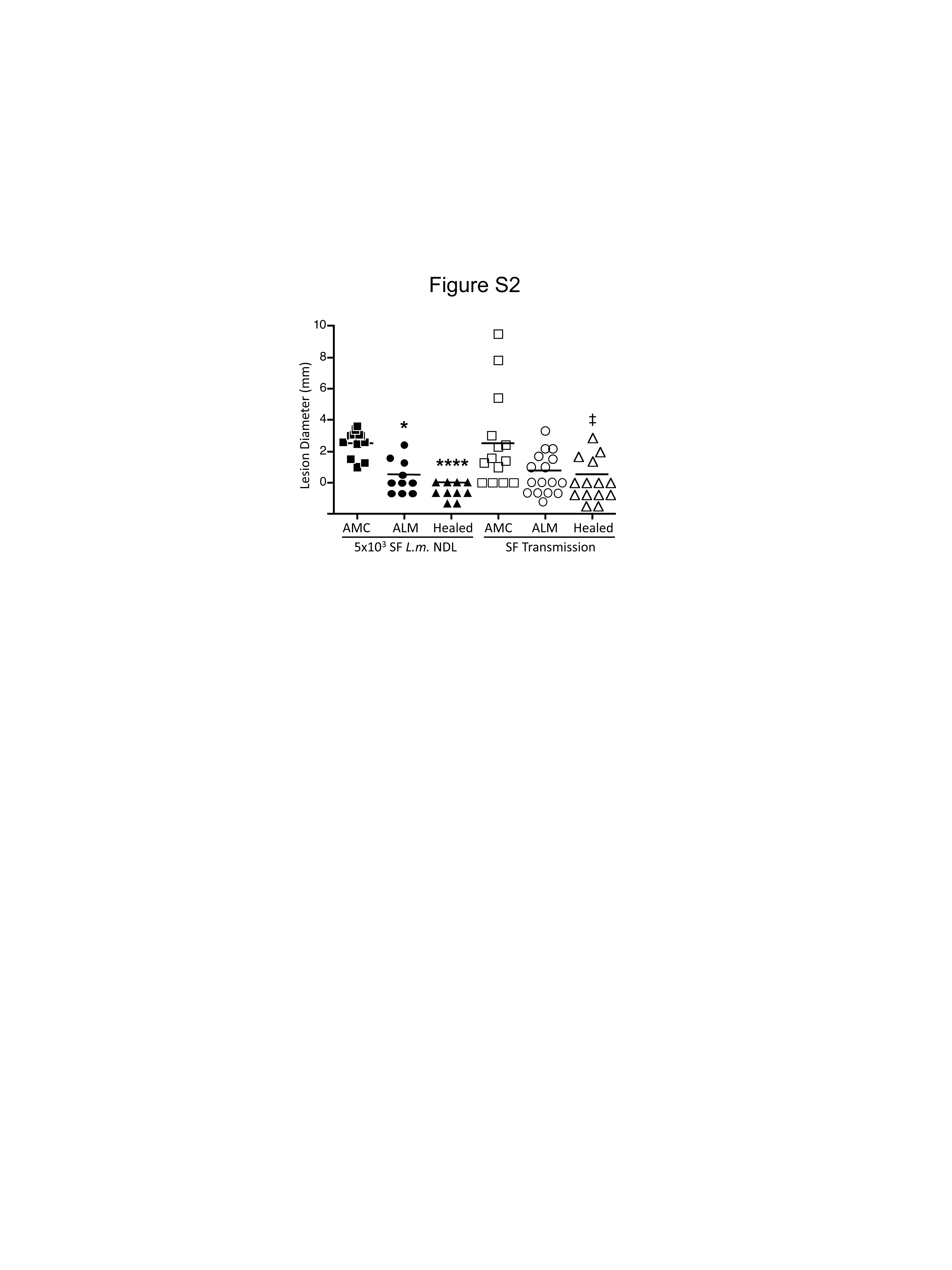

Supplement: Figure S2 — ALM+CpG vaccination reduces lesion size following needle challenge with 5×10^3^ sand fly derived L.m. metacyclic promastigotes, but not following exposure to infected sand fly challenge. Ears of AMC, ALM+CpG vaccinated (ALM), or healed mice were exposed to the bites of 4 L.m.-infected sand flies, or needle inoculated with 5×10^3^ sand fly derived L.m. metacyclic promastigotes. At 4 weeks post-challenge, the cumulative lesion diameter per ear was determined as described in materials and methods. (*) p = 0.006, (****) p<0.0001 versus AMC needle inoculated; (‡) p = 0.037 versus AMC sand fly inoculated. Lesions scores are from those ears depicted in Figure 2B. (0.39 MB TIF) [file ppat.1000484.s002.tif]

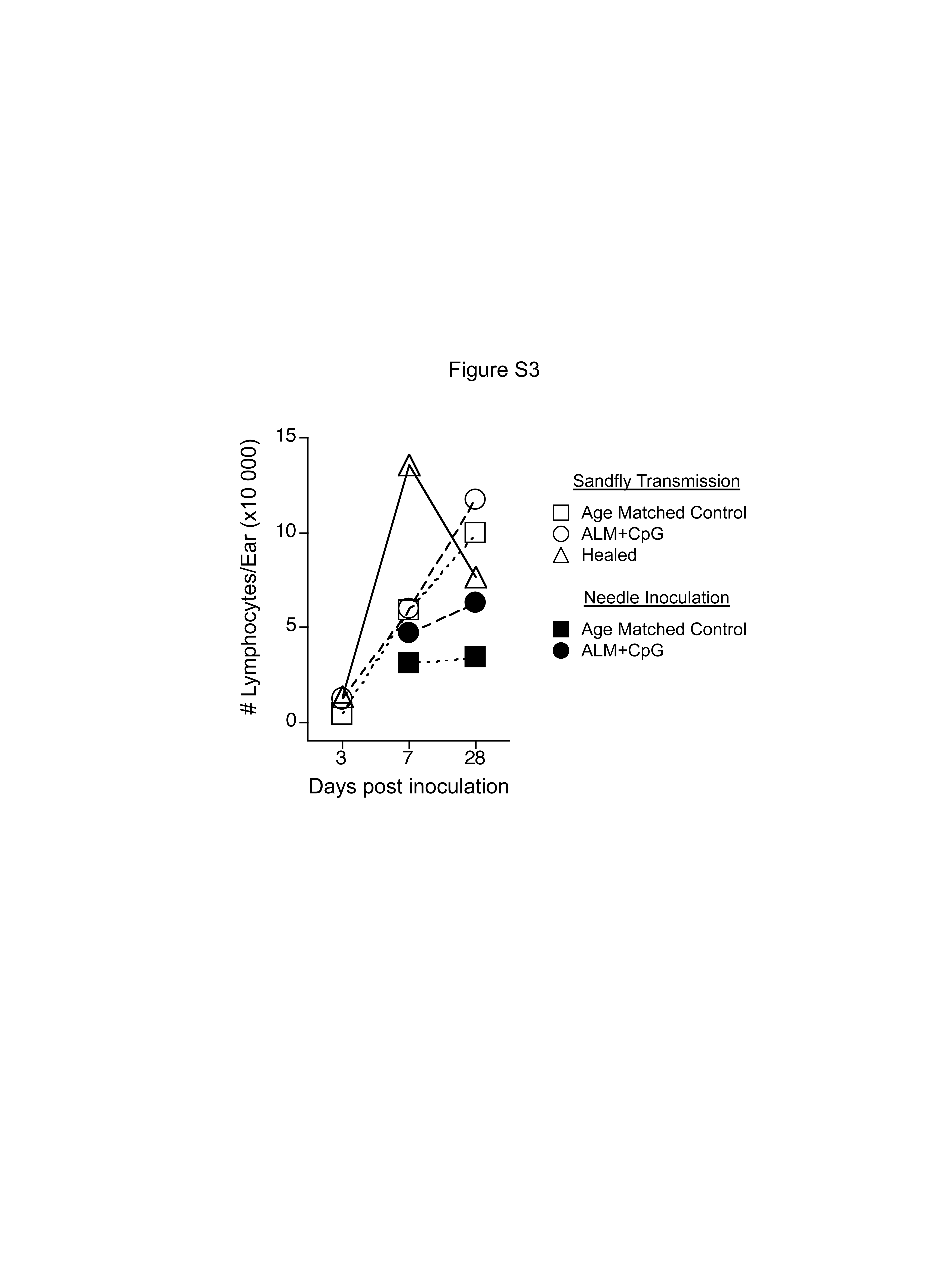

Supplement: Figure S3 — Kinetic analysis of lymphocyte recruitment to sites of needle or sand fly inoculation of L. major. Single cell suspensions of individual ears (n = 8–10) from the groups depicted in Figure 2A and Figure 3C–E, following exposure to the bites of 4 L.m.-infected sand flies (white square, Age Matched Control (AMC); white circle, Autoclaved Leishmania major (ALM)+CpG; white triangle, Healed) or needle inoculated with 103 L.m. metacyclic promastigotes (black square, AMC; black circle, ALM+CpG) were pooled, mixed 1∶1 with trypan blue, and the number of live lymphocytes per ear was determined by trypan blue exclusion and morphology. (0.45 MB TIF) [file ppat.1000484.s003.tif]
